# Characterization of bovine *FUT7* furthers understanding of *FUT7* evolution in mammals

**DOI:** 10.1186/1471-2156-13-74

**Published:** 2012-08-21

**Authors:** Benoît Laporte, Daniel Petit, Dominique Rocha, Mekki Boussaha, Cécile Grohs, Abderrahman Maftah, Jean-Michel Petit

**Affiliations:** 1 INRA UMR 1061, Unité de Génétique Moléculaire Animale, Université de Limoges, Faculté des Sciences et Techniques, 123 Avenue A. Thomas, Limoges, 87060, France; 2 INRA, UMR 1313 GABI Génétique Animale et Biologie integrative, Domaine Domaine de Vilvert, Jouy-En-Josas Cedex, 7835, France

**Keywords:** Fucosyltransferase, Sialyl-Lewis X, Evolution, Cattle, Polymorphism

## Abstract

**Background:**

The Sialyl-Lewis X (Sle^x^) is a well-known glycan structure involved in leukocyte homing and recruitment to inflammatory sites. SLe^x^ is well conserved among species and is mainly synthesized by FucT-VII in vertebrates. The enzyme responsible for its biosynthesis in cattle was not known.

**Results:**

We cloned a cDNA sequence encoding bovine α3-fucosyltransferase VII that shares 83% identity with its human counterpart. Located at the BTA 11 telomeric region, the 1029 bp open reading frame is spread over two different exons, E1 which also contains the unique 5’-untranslated region and E2 which includes the entire 3’-untranslated region. The *bfut7* expression pattern is restricted to thymus and spleen. A single transcript leading to the synthesis of a 342 aa protein was identified. The encoded fucosyltransferase, produced as a recombinant enzyme in COS-1 cells, was shown to be specifically responsible for SLe^x^ synthesis in cattle. In addition, we showed that the gene promoter evolved from fish to mammals towards a complex system related to the immune system. But beyond the fact that the gene regulation seems to be conserved among mammals, we also identified 7 SNPs including 3 missense mutations in the coding region in a small panel of animals.

**Conclusions:**

The *FUT7* sequence was highly conserved as well as the specific activity of the encoded protein FucT-VII. In addition, our *in silico* promoter analysis and the high rate of polymorphism suggested that its function is evolving toward a complex system related to the immune system. Furthermore, comparing bovine to human and mouse sequences, it appeared that a decrease in gene regulation was correlated with an increase in mutation rate and wider tissue expression.

## Background

Located at the non-reducing end of glycans comprised of glycoproteins (*N-* and *O-*linked) and glycolipids, α3-fucosylated oligosaccharides form the Lewis related carbohydrate antigen family (i.e. Le^x^, Le^y^ and Sialyl-Le^x^), one of the major components of cell surface oligosaccharides. One of them, Sialyl-Le^x^, is involved, due to its external position, in numerous cell-cell interactions occurring during embryo implantation [1], malignant transformation [2] and inflammation [3].

For example, leukocyte homing or recruitment to inflammatory and tissue injury sites involve leukocyte rolling along the endothelial wall [4,5], binding to endothelial cells near the inflammation site via integrin adhesion [6] and then migration into the underlying tissue [7]. The initial rolling step is mediated by the interaction of leukocyte glycoproteins containing active moieties such as Sialyl-Lewis^x^ with selectins expressed on endothelial cells [4]. SLe^x^ expression is constitutive on granulocytes and monocytes but not on lymphocytes [8]. Indeed, SLe^x^ expression on lymphocyte is the result of the transcriptional activation of genes coding for fucosyltransferases mainly involved in the last step of its biosynthesis.

Fucosylation is catalyzed by α3-fucosyltransferases belonging to CAZY family 10 ( http://www.cazy.org/fam/GT10.html) and especially by FucT-IV and mainly FucT-VII [9,10]. Both enzymes are Golgi resident type II glycoproteins [11,12] that catalyze the transfer of fucose in an α3-linkage from GDP-Fuc to N-acetylglucosamine of N-acetyllactosamine (Galβ1,4GlcNAc). These linkage and substrate specificities are due to the presence of 5 conserved motifs in the catalytic domain that are very important for substrate binding and the catalytic mechanism. Largely described in humans and mice [13], the α3-fucosyltransferase coding sequences are present in only one exon whereas other genes of its family generally include unstranslated exons. Furthermore, the encoded proteins are well conserved beyond species. We can distinguish two types of α3-fucosyltransferases, those with a low polymorphism rate such as FucT-IX and the others with a higher rate like FucT-III and FucT-V associated with an elevated evolution rate [14].

The aim of this paper was to assess the variability of FUT7 in mammals. In spite of its involvement in fundamental processes, known mutations in humans and mice are associated with a loss of FucT-VII enzyme activity but do not lead to critical phenotypes [15]. First we wanted to make a comparative analysis of gene structure, expression pattern among vertebrates and study the intraspecific variation of its sequence. These features are not independent as genes that evolve faster have a large number of transcripts whereas more conservative genes seem to be associated with the presence of only one transcript [16]. In contrast to other α3-fucosyltransferases genes (FUT3,4,5,6, and 9) where the coding sequence is monoexonic, the corresponding regions of human and mouse *FUT7* are split into two exons.

*FUT7* is well studied in humans and mice. Our study focused on the bovine gene since the α3-fucosyltransferase genes remain poorly documented in cattle with the exception of *futb*[17]. NCBI mapviewer website displayed a potentially encoded protein (DAA24060) 33 amino acids shorter (309 amino acids instead of 342) than that of other mammals. Surprisingly, the lacking region was localized at the end of the catalytic domain.

Here we propose a complete description of the bovine gene including its structure, transcripts and expression pattern. In addition, we characterized this sequence using phylogeny and synteny approaches as well as the function of its product by enzyme activity tests.

## Results

### Molecular cloning of potential bovine *FUT7* cDNA

**Figure 1:**
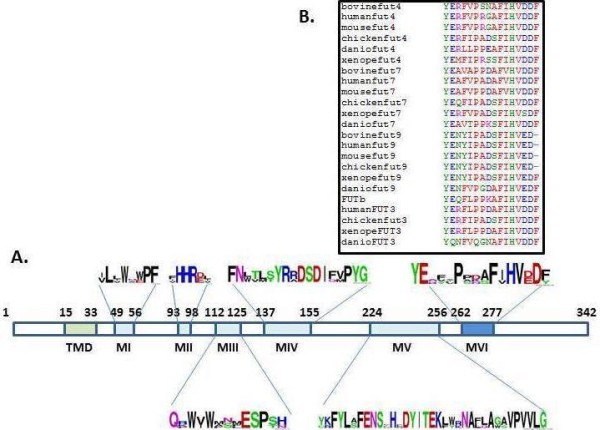
**Structure of bovine FucT-VII. A**, Bovine fucosyltransferase VII. TMD represents the transmembrane domain. MI to MVI represent the α1-3/4 fucosyltransferase specific motifs. The logos show amino acid conservation between α1-3/4 fucosyltransferases. **B**, amino acid conservation in α1-3/4 fucosyltransferase motif VI between species.

Using a partial bovine *FUT 7* gene model (generated by the Bovine Genome Sequencing Project) as a template for PCR amplification, we characterized the complete sequence of the bovine *FUT 7* gene. Thus, the coding region of cloned *bfut7* is made of 1029 bp and encodes a 342 amino acid Type II glycoprotein that respectively shares 79% and 74% of identity with its human and mouse counterparts. This protein contains the 5 specific domains of α3/4-fucosyltransferases (Figure 1) involved in substrate binding (motifs I to III) and enzyme mechanisms (motifs IV and V). Motif II possesses the HHRE sequence specific for α1/3-fucosylation activity. All motifs were strictly conserved compared to other vertebrates. Moreover, we identified a new motif (motif VI) downstream from motif V (Figure 1A) which is common to the vertebrate α3/4-fucosyltransferase family (Figure 1B).

### Characterization of bovine *fut7* and its product

**Figure 2:**
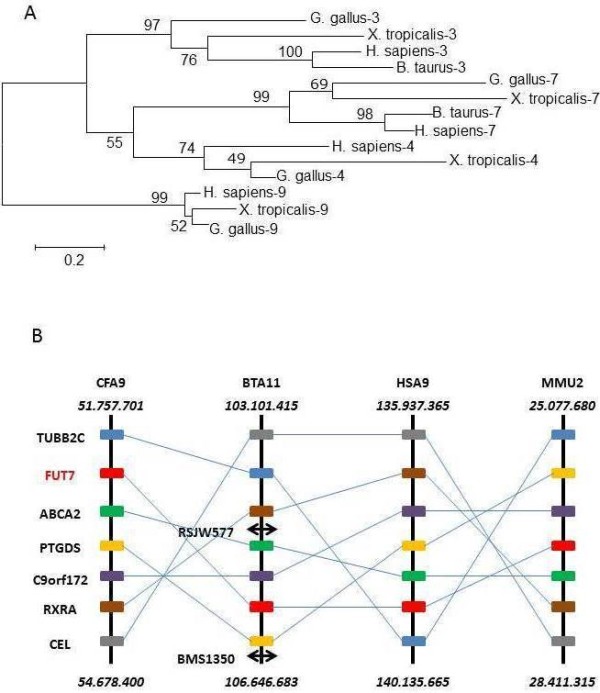
**Position of bovine FUT7 by two independent methods.** 2**A**. Molecular Phylogenetic analysis by Maximum Likelihood method based on the JTT matrix-based model [1]. The tree with the highest log likelihood (−5230.5756) is shown. The percentage of trees in which the associated taxa clustered together is shown next to the branches. A discrete Gamma distribution was used to model evolutionary rate differences among sites (5 categories (+G, parameter = 1.4694)). The rate variation model allowed some sites to be evolutionarily invariable ([+I], 14.8207% sites). The tree is drawn to scale, with branch lengths measured in the number of substitutions per site. 2**B**. Synteny shared by *Canis familiaris*, *Bos taurus*, *Homo sapiens*, and *Mus musculus* around *FUT7*. The positions of the genes (in bp) ending each chromosome segment are indicated above and below. The double arrows correspond to microsatellite markers experimentally determined in BTA11.

To assess the nature of the new bovine FUT, three independent analyzes were undertaken. Molecular phylogeny inferred by Maximum Likelihood and involving 14 amino acid sequences and 276 sites was conducted. All positions containing gaps and missing data were eliminated, and there were a total of 263 positions in the final dataset. The bovine sequence appeared to be belong to the *FUT7* group with high bootstrap values (98% with Human FUT7 and 99% with Tetrapod FUT7 (Figure 2A).

To verify the *bfut7* position in the bovine genome, we screened a bovine whole-genome radiation hybrid panel [18] by PCR. Amplification of a 278 bp genomic DNA fragment mapped *bfut7* to the telomeric region of BTA11 between *RSJW577* and *BMS1350*, two microsatellite markers respectively positioned at bases 106.193.598 and 106.646.683. *ABCA2*, *C9orf172*, and *PTGDS* genes are also found in this interval. Using Ensembl database, a synteny analysis within mammals gave a block of 6 genes surrounding *FUT7*, common to cows, dogs, mice, and humans (Figure 2B). Thus, the bovine gene has the same gene environment as other known mammalian *FUT7* genes.

To check whether bovine *bfut7* cDNA produced an active enzyme, the open reading frame was inserted into a mammalian vector and then transiently transfected in COS-1 cells. The ability of the recombinant enzyme to transfer fucose was evaluated using either sialylated or not Type I-octyl oligosaccharides, sialylated or not Type II-octyl oligosaccharides and H-Type II-octyl oligosaccharide. FucT-VII was specifically responsible for SLe^x^ biosynthesis in cattle (Table 1) because highly significant activity was mainly found with sialylated Type II substrate. In contrast, like the other tested enzymes, recombinant Fuc-TVII did not transfer fucose on Type-I substrates.

**Table 1:** **Activity of bovine** α**1-3fucosyltransferases**

**Acceptors**	**bFucT-VII**	bFucT-IV*	bFucT-IX*
**Galβ1-3GlcNAc-octyl**	0	0	0
**NeuAcα2,3Galβ1,3GlcNAc**	0	0	0
**Galβ1-4GlcNAc-octyl**	40	885	904
**Fucα1,2 Galβ1,4GlcNAc**	0	987	1068
**NeuAcα2,3Galβ1,4GlcNAc**	1108	201	43

*C.Javaud PhD (2002).

Recombinant FucT-VII, Fuct-IV* and FucT-IX were produced in COS-1 cells. Arylglycosids were used at a final concentration of 1 mM. Results are expressed as pmol fucose transferred by hour and mg proteins.

### Organization of the *FUT7* gene

**Figure 3:**
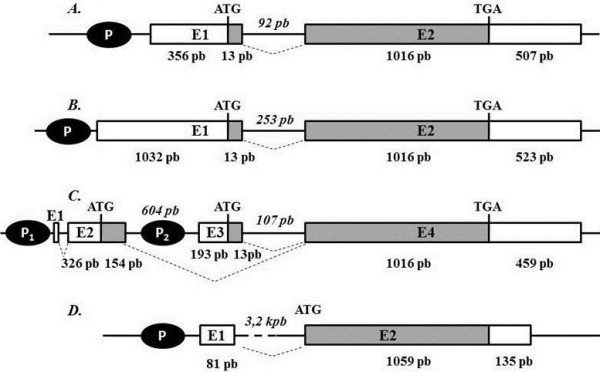
***FUT7 *gene organization. **Structure of bovine (**A**), human (**B**), mouse (**C**) and Zebrafish (**D**) genes. Grey bars represent the open reading frame while white bars indicate the gene untranslated regions. The size of exons and introns (italic) are indicated in base pairs. Dotted lines indicate a possible splicing event during the transcription. Black circles show gene promoters.

To determine gene structure, a 1135 bp fragment was amplified by PCR from genomic DNA. After sequencing, comparison with the sequence obtained from thymus cDNA showed that the ORF was comprised of 2 exons, E1 and E2. Exon E1 contained the first 13 bp of the coding sequence and E2 included the last 1016 bp of the coding region. To complete the information concerning the 3’UTR sequence, we took into account the WGS contig AAFC01684977. Taken these data together, we established that *bfut7* gene was composed of two exons E1 (369 bp) and E2 (1503 bp) including the 507 bp 3’UTR with the consensus sequence AATAAA 481 bp downstream from TGA (Figure 3A). E1 was separated from exon E2 by an intronic 92 bp sequence.

**Figure 4:**
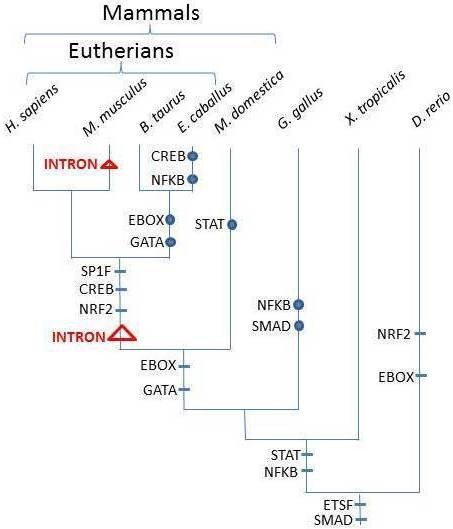
**Variation of transcription factor binding sites in *FUT7 *promotor regions in Vertebrates. **Transcription factors able to bind to the *FUT7* promotor region are placed on the branches of the phylogenetic tree (horizontal bars) according to their appearance. Circles indicate the loss of DNA sites to which the transcription factors can bind. Triangles point out the split of *FUT7* due to the insertion of intronic sequences into the coding region.

When comparing *FUT7* gene organization, it appears that in gray short-tailed opossum (*Monodelphis domestica*), chicken (*Gallus gallus*) and western clawed frog (*Xenopus tropicalis*), the whole coding sequence is included in only one exon that corresponds to the gene sequence. A similar organization is observed in zebrafish (*Danio rerio*) with the exception of an upstream short untranslated exon (Figure 3D). In Eutherians, the coding sequence is split into two exons. In mammals, the emergence of the intron occurred at the base of Eutherians (Figure 4), since we identified an intronic sequence, similar to those observed in human and cattle (Figure 3A and B), in *Loxodonta africana* (data not shown) whereas no introns were retrieved in *M. domestica*. The main exon has always the same coding size (1016 bp). It should be noted that the ancestor of rats and mice gained another intron upstream from the previous one (Figure 3C).

### *bfut7* expression in different bovine tissues

**Figure 5:**
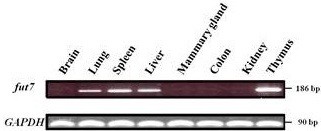
***bfut7 *gene expression in different bovine tissues. ** The gene expression pattern was determined by RT-PCR using mRNA from 8 different bovine tissues. Forward primer (*fut7f*) was chosen in exon E1 and reverse primer (*fut7r*) in exon E2 in order to amplify a 186 bp specific cDNA fragment. A *GAPDH* fragment of 90 bp was amplified as a control.

In order to determine the *bfut7* expression pattern, we performed a RT-PCR using forward and reverse primers localized in two different exons, respectively exons 1 and 2, to avoid genomic DNA contamination. Among the eight tested tissues, we detected *bfut7* transcripts in thymus, spleen, liver and lung (Figure 5). It was most highly expressed in the thymus and was lower in the lung. Finally, lung is the tissue where the gene expression is the weakest. Conversely, we did not detect any *bfut7* transcripts in brain, mammary gland, colon and kidney. Moreover, 5’-RACE analyses done in tissues expressing the gene revealed a single transcript with a 356 base 5’-UTR.

In connection with tissue expression, *in silico* searches for TFBS (transcription factor binding sites) were undertaken as the pattern of the bovine gene expression seemed to be more widespread than its human and mouse counterparts. Thus, during vertebrate evolution, it seems that two TFBS, i.e., ETSF and SMAD, have been conserved among the studied species (Figure 4). EBOX could be also considered as ancestral in vertebrates but a greater number of sequences should be added to test this alternative model. The number of transcription factors able to bind in the 1000 bp upstream from the first exon increases with species evolution. Indeed in most Eutherians, the *FUT7* expression seems to be regulated by at least nine transcription factors, five of them being common to humans, mice, horses and cattle. However, the bovine ancestor has probably lost two of them (EBOX and GATA). In other vertebrates, a lower amount of transcription factor binding sites has been found and varies from 2 in *Gallus gallus* to 4 in *Danio rerio*.

### Intraspecific variation of *bfut7*

Since mutations have been described in humans and mice to lead to the loss of enzyme activity and inflammatory diseases, we investigated gene variations by sequencing genomic DNA from 30 animals belonging to six different cattle breeds (Blonde d’Aquitaine, Charolaise, Holstein, Limousine, Montbéliarde and Normande). We identified seven SNPs in the coding region of *bfut7* at bases 58, 95, 213, 335, 501, 792 and 831 (Table 2). Among these mutations, three were missense (58, 95 and 335). At position 58, substitution of a guanine by an adenine induces the translation of a methionine instead of valine 20. At positions 95 or 335, the SNP G→A changes R_32_ or R_112_ in Q. Surprisingly, in the five Holstein animals, no polymorphisms were found while in the other breeds we always identified mutations in spite of the relatively low number of samples.

**Table 2:** **Polymorphism analysis of *bfut7 *in cattle breeds**

	**Haplotype**							**Number of animals with the polymorphic sequence**
	**Base position in the coding sequence**							
**Breeds**	**58**	**95**	**213**	**335**	**501**	**792**	**831**	
Holstein*	G/G	G/G	C/C	G/G	G/G	C/C	C/C	-
**Blonde d'Aquitaine**	**A**/G	-	-	-	-	-	-	3
	**A**/G	-	-	-	-	-	C/**T**	1
	-	-	-	**A**/G	-	-	-	1
**Charolaise**	-	-	C/T	A/G	-	-	-	1
	-	-	T/T	A/A	-	-	-	2
	A/A	A/A	-	-	-	-	C/T	1
**Limousine**	-	-	-	-	G/C	G/C	-	1
	-	-	-	A/G	-	-	-	1
	-	-	C/T	A/G	-	-	-	1
**Montbéliarde**	-	-	T/T	-	-	-	-	1
	-	-	T/T	A/A	-	-	-	1
	-	-	C/T	A/G	-	-	-	2
	-	-	C/T	-	-	G/C	-	1
**Normande**	-	-	C/T	-	-	-	-	1
	A/G	-	C/T	-	-	-	-	1
	-	-	C/T	A/G	-	-	-	2
	-	-	T/T	A/G	-	-	-	1
**Position of amino acid and substitution**	**20 aa V → M**	**32aa R → Q**	**38 aa None**	**112 aa R → Q**	**167 aa None**	**264 aa None**	**277 aa None**	

Data were obtained from 6 cattle breeds. (Blonde d’Aquitaine, Charolaise, Limousine, Holstein, Normande and Montbéliarde). For each breed, 5 animals were analysed. * The haplotype found for the Holstein breed is also that of the reference sequence (DQ339142.1).

## Discussion

Terminal fucosylation is involved in a large number of processes such as cell migration and leukocyte recruitment to inflammatory sites [10]. This process implies the presence of a specific glycan structure, sialyl-lewis X mainly synthesized by the Fuc-TVII enzyme. The gene encoding for this protein, *fut7*, is widespread among other vertebrate species [14]. To date, no evidence for an active glycosyltransferase synthesizing the sialyl-lewis X motif has been identified in cattle although the structure is present [19], and surprisingly the reference sequence in NCBI data bank corresponds to an enzyme truncated in its C-terminal catalytic domain. In this study, we describe the bovine gene, *bfut7* that encodes an active enzyme responsible for synthesis of sialyl-Lewis X motif.

This gene spans two exons separated by a short intronic sequence (Figure 3) and is located in the telomeric region of BTA11, a region homologous to HSA9, MMU2 and CFA9 (Figure 2) which contains the human, mouse and dog *FUT7* gene [20], supporting UMD3.1 assembly [21]. Unexpectedly, this location is not related to a known QTL associated with resistance to diseases such as mastitis [22] whereas its product recognized mainly sialyl type II as a substrate and thus elaborated the sialyl-lewis X motif (Table 1) involved in leukocyte recruitment.

Only one transcript containing both exons was found in tested tissues. The expression pattern was similar to that observed in other mammals for thymus and spleen [23] whereas its expression in lung and liver seems to be a bovine specificity. Indeed, the hypothesis of lung expression due to the presence of leukocytes is unlikely because the individuals were considered as healthy by animal control. Until now, *FUT7* expression in lungs has not been described in lung epithelial cells, and when a sialyl-Lewis X motif was observed at these cell surfaces in humans, it was correlated to *FUT4* expression [24]. Liver expression was described in human liver, but only in particular cases such as liver regeneration [2]. In mice, only one EST from liver is found in the Unigene database.

We also observed a change in gene organization associated with the evolution of the promoter region. The monoexonic structure including coding and 5’and 3’ untranslated sequences found from *D. rerio* to *G. gallus*, was split in Eutherians (bovine and human) into at least two exons, one of them bearing a short coding sequence. In contrast, other α1,3/4 fucosyltransferase genes described in vertebrates only have a monoexonic coding sequence [25]. Exon 1 possesses the 13 first nucleotides of the coding sequence in both species (Figure 3), also found in mice and rats, but corresponding to exon 3. In these species, another exon (E2) contains a 154 bp sequence coding for the first Fuc-TVII amino acids, and leads to an alternative longer form (52 amino acids) of the protein [26]. This is the result of alternative splicing and from the use of two promoter regions that generates 4 transcripts while only one is found in other vertebrate *fut7* genes. A search for putative transcription factor binding sites showed that in mice two regions contain the sites, one upstream from E1 and corresponding to the long form of the protein and another upstream from the 67 bp E3 giving rise to the same protein as observed in humans and cattle. Only one binding site for E-Twenty Six Factor (ETSF) was found in all species studied from *D. rerio* to *H. sapiens*. It has been described to participate in differentiation, maintenance and function of the immune system [27]. A greater number of transcription factors seemed to regulate *FUT7* transcription in *M. musculus*, *B. taurus* and *H. sapiens* (Figure 4). This may be explained by cis-clusters including ETSF, SP1F, STAT, EBOX and CREB that occur in activated T-cells, and by CREB, NFKB and GATA that are involved in stimulated lymph nodes [27]. It has been also shown that CREB in association with another transcription factor regulates the activation and proliferation of macrophages [28], and that the SMAD signaling pathway plays a role in hematopoiesis and translational hematology [29]. A binding site for GATA was observed only in humans and mice. This transcription factor regulates the cell-lineage-specific expression of lymphocyte homing receptors [30]. These data strongly suggest that the *FUT7* promoter region became more complex and could related to the immune system evolution in vertebrates and with the phylogenic tree of Fuc-TVII [14]. Surprisingly, no significant binding sites for the last cluster have been described in horses and cattle suggesting another type of regulation of the *fut7* gene in lymph nodes for these species.

**Figure 6:**
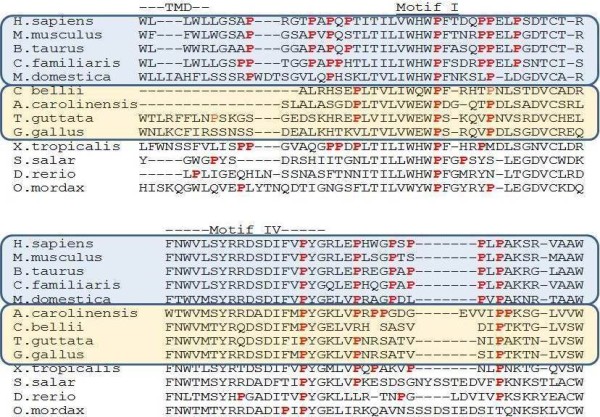
**Alignment of the region surrounding the FucT-VII motifs I and IV in mammals, birds, lizards, amphibia and fish.** Prolines are in red characters and position of transmembrane domain (TMD) and Motif I are indicated. Mammals are boxed in blue and include humans (*Homo sapiens*; AAH86312), mice (*Mus musculus*; NM_013524), cows (*Bos taurus*), dogs (*Canis familiaris*; NP_001005379) and possums (*Monodelphis domestica*; XP_001374334). The Sauropsidae are boxed in orange and include Carolina lizards (*Anolis carolinensis*; WGS AAWZ02039323.1 Cont2.39322), Painted turtles (*Chrysemys picta bellii*; WGS AHGY01431622.1 Contig440.209), chickens (*Gallus gallus*; BAB82491) and Zebra-finch (*Taeniopygia guttata*; XP_002192597). The last four sequences correspond to the western clawed frog (*Xenopus tropicalis*; AY692026), Atlantic salmon (*Salmo salar*; NP_001133942.1), zebrafish (*Danio rerio*; AY788991), and rainbow smelt (*Osmerus mordax*; ACO08841).

Amino acid sequence alignment of vertebrate Fuc-TVII shows a strong identity within the five conserved motifs previously described [31,32]. However, compared to Fuc-TIV and Fuc-TIX, two enzymes implicated in lewis X motif synthesis, this identity is lower since between vertebrate species, 98% homology was observed for Fuc-TIX [23]. For Fuc-TVII the larger differences are observed near motifs I and IV, respectively involved in substrate binding and enzyme activity. Indeed, the amount of proline residues is especially high in mammals (Figure 6) and maybe correlated to a change in specification of acceptor substrates. It should be interesting to further investigate the role of these numerous prolines in *Xenopus tropicalis* motif I and *Anolis carolinensis* motif IV (Figure 6).

In the region implicated in the catalytic mechanism after motif V, we identified a new motif (motif VI) containing 16 amino acids. A short and well conserved sequence HVD/ED was observed at the end of this motif. Glutamic acid residue was found in Fuc-TIX, an enzyme that synthesizes mainly Lewis X and Lewis Y (Table 1) whereas an aspartic acid residue was found in the other enzymes that synthesize sialyl-Lewis. bFuc-TVII produces only sialyl-Lewis X and, like its human and mouse counterparts, preferentially recognizes Sialyl-LacNAc as a substrate. We suggest that the presence of aspartic acid in this short sequence leads to preferential recognition of sialylated acceptor substrates.

Fuc-TIV and Fuc-TIX are implicated very early in development [33,34] and until now only a very small number of mutations have been described for *FUT9* in data bases. This precludes its crucial function. In contrast, in humans, a large number of mutations has been described for *FUT7* with a frequency around 1%. Among them, one induces the loss of enzyme activity associated with the appearance of diseases such as arthritis [16]. In bovines we identified seven polymorphisms after sequencing the *bfut7* coding sequence in 30 unrelated cattle. Three were missense mutations. Surprisingly, no mutations were found in a diary breed (Holstein), whereas they were detected in beef breeds (Blonde d’Aquitaine and Limousine) and in dual-purpose breeds (Montbéliarde and Normande). More intense selection may have further reduced levels of genetic variation in Holsteins [35] and could explain the absence of polymorphism in this breed. Compared to humans and mice, a high polymorphism rate between animals from the same breed without notable changes in phenotype could be related to the greater number of tissues where *bfut7* is expressed. In bovine, the encoded enzyme could act on a richer protein repertoire. This specificity could have consequences for modulation of the immune response, known to be complex in bovines, required for control of the substantial load of microorganisms present in the rumen and close contact in the herd that promotes rapid disease transmission [36].

## Conclusions

We can conclude that the *bfut7* gene encodes an enzyme that mainly ensures the synthesis of the sialyl-lewis X motif. Regulation of its expression seems to be more and more specific and complex from fishes to mammals. Indeed, in mammals, expression mainly depends on transcription factors associated with the immune system response. Whereas its implication in leukocyte recruitment is well documented, the relatively large amount of polymorphism depicted in few animals correlated well with a gene extending its abilities in terms of tissue expression and target proteins in bovines.

## Methods

### Materials

Oligonucleotides were synthesized by MWG (Germany). The 2X pre-aliquoted PCR master mix was from Abgene. GDP- [^14^C]Fucose (310 mCi/mmol) was from Amersham Pharmacia Biotech (USA). Dulbecco’s modified Eagle’s medium (DMEM) with 4.5 g/L glucose and fetal calf serum were from Eurobio (France). L-Glutamine, nonessential amino acids, antibiotics and trypsin-EDTA 1X were from Invitrogen Life Technologies (France). Sialyl-type I and II oligosaccharides were provided by Lectinity (Russia).

### Molecular cloning of bfut7 cDNA

A WGS contig (AAFC01684977) was identified by BLAST analysis of the bovine genome sequence databases with human *FUT7* (U11282) and mouse *fut7* (AAC52484) mRNA sequences. High similarities were found with the last 410 bp of the coding region and oligonucleotide primers were designed, *fut7f*_*1*_ and *fut7r*_*1*_ (Table 3). Moreover, a primer whose sequence recovers a highly conserved region in human and mouse was also designed (*fut7f2*). For amplification of the bovine *fut7* sequence, total RNA were extracted from 250 μg bovine tissues with the Qiagen RNeasy Midi kit. Then, a reverse transcription was performed with 10 μg of total RNA using the High capacity cDNA Archive Kit (Applied Biosystem, Foster City, CA) with random primers. From the obtained cDNA, PCR amplifications were done using primers *fut7f*_*1*_ or *fut7f2* and *fut7r*_*1*_ (Table 3). The 25 μL PCR mixture contained 1 unit of DyNazyme EXT, 0.4 μM of each primer, 0.2 mM of dNTP, 0.2 μg of cDNA and 10% DMSO (v/v). Reactions were run under the following conditions: a first denaturation step of 7 min at 94°C followed by 40 cycles, each of them including 1 min at 95°C, 1 min at 62°C, 2 min at 72°C, and a final extension of 7 min at 72°C. The whole coding region was obtained after 5’-RACE analysis which was run following the instructions of the 5’/3’RACE Kit, 2^nd^ generation (Roche, Mannheim, Germany). For that, primer *fut7r*_*3*_ was used for the total RNA reverse transcription and primer pairs *OligodT-Anchor primer/fut7r*_*4*_ and *PCR anchor primer/fut7r*_*5*_ (Table 3) were used for the first and nested PCR respectively. All PCRs were performed with 8% DMSO (v/v). PCR programs were conducted at an annealing temperature of 60°C. All PCR products were subcloned into the Topo TA sequencing vector (Invitrogen, Carlsbad,CA). Sequencing reactions were run with the Big Dye Terminator v1.1 kit (Applied Biosystem) on a 3100 genetic analyzer (Applied Biosystem). Sequence comparisons were done using Align Sequence Nucleotide BLAST on the NCBI site using the bovine *fut7* sequence DQ339142.1 as a reference.

**Table 3:** Oligonucleotide primers used in this paper

**Name**	**Primer sequence**
*fut7f*_*1*_	5’-CCTGTGCGCCAGCTGCCTTCTG–3’
*fut7f*_*2*_	5’-CACCATCCTTGTCTGGCACTGG-3’
*fut7f*_*3*_^*(a)*^	5’-GA**GAATTC**AGGAATGCAGAATGCTGG-3’
*fut7f*_*4*_	5’-GCCGTGAATGCAGAATGCTGG-3’
*fut7r*_*1*_	5’-GCAGCGCTCAGGCCTGGAACC-3’
*fut7r*_*2*_	5’-CAGAAGGCAGCTGGCGCACAGG-3’
*fut7r*_*3*_	5’-GCTCAGCCAGGGGCGGGCG-3’
*fut7r*_*4*_	5’-GGCGTCGGCGCCGGCCAGCAGG-3'
*fut7r*_*5*_	5’-GGCTGGCTGGCAAATGGCCAGTGCC-3’
*fut7r*_*6*_^*(b)*^	5’-CT**GGTACC**CACAGCTCAGGCCTGGA-3’
*Oligo d(T)-Anchor primer*	5’-GACCACGCGTATCGATGTCGACTTTTTTTTTTTTTTTTV-3’
*PCR Anchor primer*	5’-GACCACGCGTATCGATGTCGAC-3’
*GAPDHf*	5’-GTGAAGCAGCGCTCAGAGG-3’
*GAPDHr*	5’-TTGAAGTCGCAGGAGACAACC-3’

*in bold restriction site (**a**) *Eco*RI and (**b**) *Kpn*I.

### Tissue expression, organization and chromosome localization of *bfut7*

Tissues were collected from Limousine cattle at the slaughterhouse. The presence of *fut7* transcripts in bovine tissues was detected by PCR using cDNA, prepared as described in *Molecular cloning of the bfut7 cDNA*, under the following conditions: 12.5 μL pre-aliquoted 2X PCR master mix (Abgene, Courtaboeuf, France), 0.3 μM primers (*fut7f*_*4*_*/fut7r*_*5*_; Table 3), 0.2 μg cDNA and 10%DMSO (v/v) in a final reaction volume of 25 μL. The PCR program included the following steps: 3 min of denaturation at 94°C, then 45 cycles of 1 min at 94°C, 60°C for 30 s, and 45 s at 72°C followed by a final extension step of 3 min at 72°C. For *GAPDH*, primer pair *GAPDHf/GAPDHr* (Table 3) was used at 0.3 μM in a 25 μL reaction including 12.5 μL pre-aliquoted 2X PCR master mix (Abgene) and 0.2 μg cDNA. The cycling conditions included an initial incubation at 94°C for 3 min followed by 37 cycles of 30 s at 94°C, 30 s at 56°C, and 30 s at 72°C and then a final extension step at 72°C for 5 min. Touchdown PCR conditions were used. The annealing temperature was decreased from 62°C to 56°C and so by the loss of 1°C/cycle during the seven first rounds of the PCR. The annealing temperature was then maintained constant at this temperature for the next cycles.

Same PCR reactions using primer pair *fut7f*_*4*_*/fut7r*_*5*_ (Table 3) were performed on 0.1 μg genomic DNA to determine gene structure or to screen the whole genome radiation hybrid panel described by [17] to determine *bfut7* chromosome localization.

### Polymorphism analysis

Polymorphism was examined in 30 animals coming from 6 different cattle breeds (Blonde d’Aquitaine, Charolaise, Holstein, Limousine, Montbéliarde and Normande). Five animals were taken per breed. Blood from these animals was collected at the slaughterhouse. Genomic DNA was extracted from blood cells using QIAGEN-DNeasy Blood and Tissue Kit. Then, fut7 was amplified as 3 fragments using primer pairs, *fut7f*_*4*_*/fut7r*_*5*_, *fut7f*_*1*_/*fut7r*_*1*_, *fut7f2*/*fut7r*_*1*_ under PCR conditions described in molecular cloning of *bfut7* gene. The PCR products were then sequenced with each primer using the Big Dye Terminator v1.1 kit (Applied Biosystem) on a 3100 genetic analyzer (Applied Biosystem).

### Expression of recombinant bFucT-VII and fucosyltransferase assays

A DNA fragment including the complete coding sequence was amplified by PCR from thymus cDNA using primers *fut7f*_*3*_ and *fut7r*_*6*_ and the same PCR conditions described in molecular cloning of *bfut7* cDNA. The fragment was then cloned into the pFlag CMV-2 mammalian expression vector (Sigma) between *Eco*RI and *Kpn*I restriction sites. COS-1 cells were transfected with 6 μg native or recombinant expression vector with FuGENE 6 Transfection Reagent (Roche). After 48 h, cells were trypsinized and proteins were extracted from pelleted cells with a lysis buffer containing 10 mM sodium cacodylate, pH6, 20% (v/v) glycerol, 1 mM DTT and 1% (v/v) Triton X100.

Enzyme assays were performed in 50 μl containing 25 mM sodium cacodylate pH 6.5, 5 mM ATP, 20 mM MnCl_2_, 10 mM α-L-fucose, 3 μM GDP-[^14^C]-fucose (310 mCi/mmol), 0.1 mM acceptor substrate. After 2 h incubation, reactions were stopped by adding 1 ml water and applied to a conditioned Sep-Pak C_18_ reverse chromatography cartridge (Millipore Corp., MA). After a 15 ml H_2_O wash, products were directly eluted by 10 ml of methanol into scintillation vials and counted with one volume of Instagel (Packard, IL) in a liquid scintillation beta counter (TRI-CARB 2100 TR, IL).

### Bioinformatics

To identify putative promoter sequences within a 1000 bp region upstream from the start codon, genomatics software tools were used ( http://www.genomatics.org), applying a threshold of 94% similarity. Sequences were extracted from NCBI databases (*Equus caballus*: NW_001867391.1, *Homo sapiens*: NW_001839245.1, *Mus musculus*: NW_001030686.1, *Didelphis domestica* : traces gn1|ti|474330053 and gn1|ti|526972938, *Bos taurus* gi|269932530:97318–108050, *Xenopus tropicalis* Ensembl scaffold:JGI_4.2:GL173522.1:48484:50721:-1 and *Gallus gallus* gi|118099516:288657–388656). The access numbers for Fuc-TIV, Fuc-TIX and Fuc-TIII in western clawed frog, chicken, and human were the same as the previous paper on vertebrate FUT phylogeny [14]. Alignments of 14 amino acid sequences were obtained using the program MUSCLE (Multiple Sequence Alignment) available at: http://www.ebi.ac.uk/Tools/msa/muscle/. Among the 576 sites, uninformative positions mainly contained in the stem region of the molecule were removed, and 276 were retained.

Phylogeny was conducted through the Maximum Likelihood method implemented in MEGA5.0 [37]. The best choice of substitution pattern between amino acids [38] followed the calculation of BIC (Bayesian Information Crirerion). The robustness of branches was tested with 1000 bootstrap replicates.

## Competing interests

We formally declare that this manuscript and its contents do not have any competing interest.

## Authors' contributions

BL and JMP conceived the study and wrote the paper, BL carried out most experimental work, DR performed polymorphism analyses, MB and CG determined chromosome localization, DP contributed to bioinformatics, AM, DR and DP provided substantial editorial advice, and all authors have read and approved the final manuscript.
